# Roles of ERβ and GPR30 in Proliferative Response of Human Bladder Cancer Cell to Estrogen

**DOI:** 10.1155/2015/251780

**Published:** 2015-05-18

**Authors:** Weiren Huang, Yuanbin Chen, Yuchen Liu, Qiaoxia Zhang, Zhou Yu, Lisha Mou, Hanwei Wu, Li Zhao, Ting Long, Danian Qin, Yaoting Gui

**Affiliations:** ^1^Guangdong and Shenzhen Key Laboratory of Male Reproductive Medicine and Genetics, Institute of Urology, Peking University Shenzhen Hospital, Shenzhen PKU-HKUST Medical Center, Shenzhen 518036, China; ^2^Key Laboratory of Medical Reprogramming Technology, Shenzhen Second People's Hospital, First Affiliated Hospital of Shenzhen University, Shenzhen 518035, China; ^3^Department of Physiology, Shantou University School of Medicine, Shantou 515031, China; ^4^Department of Physiology, Medical College of Jiaying University, Meizhou 514031, China; ^5^Anhui Medical University, Hefei 230032, China; ^6^The Institute of Plastic Surgery, Xijing Hospital, Fourth Military Medical University, Xi'an 710032, China

## Abstract

Bladder cancer belongs to one of the most common cancers and is a leading cause of deaths in our society. Urothelial carcinoma of the bladder (UCB) is the main type of this cancer, and the estrogen receptors in UCB remain to be studied. Our experiment aimed to investigate the possible biological effect of 17*β*-estradiol on human bladder-derived T24 carcinoma cells and to indicate its related mechanisms. T24 cells were treated with various doses of 17*β*-estradiol, and cell proliferation was detected using MTT assays. 17*β*-estradiol promoted T24 cell proliferation independent of ER*β*/GPR30-regulated EGFR-MAPK pathway, while it inhibited cell growth via GPR30. Furthermore, the expression levels of downstream genes (*c-FOS, BCL-2,* and *CYCLIN D1*) were increased by 17*β*-estradiol and this effect was independently associated with activity of the EGFR-MAPK pathway. The two estrogen receptors might be potential therapeutic targets for the treatment of bladder cancer.

## 1. Introduction

Bladder cancer is currently the fourth most common cancer worldwide and accounts for a high number of deaths every year [1]. It is widely acknowledged that sex hormones exert a complicated function* in vivo*. Previous studies showed that estrogens play important roles in the initiation and proliferation of bladder cancer through specific receptors-induced signaling pathways [2–5]. However, reports also showed that females who are treated with estrogens have reduced risk of bladder cancer [6, 7], implying that estrogens may contribute to the prevention of bladder cancer. Estrogens exert their biological function primarily through binding to estrogen receptors (ERs), which include the classic nuclear ERs (ER*α* and ER*β*) [8] and/or the membrane ERs [9]. ER*α* is rarely expressed in bladder cancer cells [5, 10], while ER*β* is expressed at high levels in both normal urothelial and bladder cancer cells [5]. Furthermore, it is considered that ER*β* expression is abundant in cases of both low-grade and high-grade cancers [5], implying that ER*β* plays important roles in bladder cancer.

GPR30 (G protein-coupled receptor 30), a novel membrane ER with high-affinity and low-capacity binding to estrogens, is structurally dissimilar to nuclear ERs [11] and localizes to both the plasma membrane and endoplasmic reticulum [12, 13]. GPR30 has been detected in multiple tumors and plays important roles in cell proliferation and differentiation [14–17]. Activation of GPR30 results in inhibition of prostate cancer PC-3 cell proliferation [16] and stimulation of testicular germ cell-JKT-1 cell proliferation [17]. These effects are probably not induced by the same signaling pathways.

Several studies have investigated the effects mediated by ERs [4, 5, 18] and GPR30 in bladder cancer [19]; however, the observations were controversial. In addition, few studies have explored the function regulated by the two ERs subtypes. In this study, we aimed to elucidate the biological action of 17*β*-estradiol (E2, <1 *μ*mol/L) on bladder cancer* in vitro* and to investigate the involved mechanisms. As 90% of the cases of bladder cancer are transitional cell carcinoma (TCC) [20], we used T24, a human bladder transitional cell carcinoma line, as an experimental model.

## 2. Materials and Methods

### 2.1. Cell Culture

T24 human carcinoma cells (ATCC HTB-4) were cultured at 37°C with 5% CO_2_ in RPMI 1640 medium (Gibco-BRL, Grand Island, NY, USA) supplemented with 10% dextran-coated charcoal-treated fetal bovine serum (FBS; Hyclone, UT, USA) and 100 U/mL penicillin and streptomycin. Cells were plated in 6-well plates at a density of 1 × 10^5^ cell/well. The experimental reagents were added to fresh phenol-red-free RPMI 1640 medium after one night of serum starvation. After specific treatment times, the exponentially proliferating cells in this study were used for quantitative real time PCR and western blotting analyses.

### 2.2. MTT Assays

To observe the effect of E2, T24 cells were seeded in 96-well plates at a density of approximately 2 × 10^3^ cells/well. Then E2 or E2-BSA was added at final concentrations of 0.1 nM, 1 nM, 10 nM, 100 nM, or 1 *μ*M, and 0.1% DMSO was used as the basal control group. Cells were treated in quadruplicate for each condition. After the cells were incubated for 0, 24, 48, 72, and 96 h, 20 *μ*L 3-(4,5-dimethythiazol-2-yl)-2,5-diphenyltetrazolium bromide (MTT) (Sigma-Aldrich, St. Louis, MO) solution [5 g/mL in phosphate buffered saline (PBS)] was added to each well. The cells were incubated at 37°C for 4 h; then media were removed and 150 *μ*L dimethyl sulfoxide (DMSO) was added per well to solubilize the formazan. The microplate was shaken on a rotary platform for 10 mins at room temperature, and then the optical density (OD) values were measured at 490 nM using a Wellscan reader (Bio-Rad Laboratories, Hercules, CA, USA). To investigate the signaling pathways activated by E2, T24 cells were pretreated with specific siRNA or inhibitors prior to E2 addition, and the results were examined as described above. The cell inhibition rate = (control group value − experimental group value)/control group value × 100%. Three dependent experiments were performed. The data presented here was from one representative experiment.

### 2.3. Quantitative PCR

Total RNA from T24 cells was extracted using TRIzol reagent (Invitrogen, Carlsbad, CA, USA) and reverse transcribed using PrimeScript RT Kit (Takara, Shiga, Japan). We determined the expression of* c-FOS*,* BCL-2*,* CYCLIN D1*, and *β-actin* using the ABI PRISM 7000 instrument (ABI, CA, USA). The primers were as follows:

 
*c-FOS* (forward, 5′-AGGAGAATCCGAAGGGAAAG-3′; reverse, 5′-CAAGGGAAGCCACAGACATC-3′),
 
*BCL-2* (forward, 5′-GGGAGGATTGTGGCCTTCTT-3′; reverse, 5′-ATCCCAGCCTCCGTTATCCT-3′),
 
*CYCLIN D1* (forward, 5′-CATGGAAGCGAATCAATGGACT-3′; reverse, 5′-CCTCCTTCTGCACACATTTGAA-3′),
 
*β-actin* (forward, 5′-CTGGAACGGTGAAGGTGACA-3′; reverse, 5′-AAGGGACTTCCTGTAA-3′).

The PCR cycling parameters were denaturation at 95°C for 30 s, followed by 40 cycles at 95°C for 5 s and 60°C for 30 s.

### 2.4. Western Blotting

T24 cells exposed to reagents in 6-well plates were lysed in 200 *μ*L RIPA buffer (Invitrogen, Carlsbad, CA, USA), which contained a final concentration of 1 mM NaF (Sigma-Aldrich, St. Louis, MO) and 1 mM Na_3_VO_4_ (Sigma-Aldrich, St. Louis, MO), and cells were then sonicated on ice for 10 s. After centrifugation at 12,000 ×g for 10 min, the supernatant was transferred to a clean Eppendorf tube and then boiled at 100°C for 5 min in loading buffer containing mercaptoethanol. The whole proteins (20 *μ*g) extracted from each sample were resolved on a gradient SDS-PAGE gel and electrotransferred onto PVDF membranes (Millipore, Billerica, MA) using a wet transfer cell (Bio-Rad Laboratories, Hercules, CA) at 200 mA for 2 h. Membranes were preblocked in Tris-buffered saline containing 0.1% Tween 20 and 5% BSA (TBST-BSA) and then were incubated with phospho-ERK-specific antibodies (Cell Signaling Technology, Beverly, MA, USA) diluted at 1 : 1000 in TBST-BSA overnight at 4°C, followed by species-specific HRP-conjugated secondary antibodies (KPL, Gaithersburg, MD, USA) diluted at 1 : 2500 in TBST-BSA for 1 h at room temperature. Blots were developed using ECL procedures. Relative expression levels of total ERK protein in each sample were determined by stripping the phospho-ERK-specific antibodies from the membranes and reincubating with ERK antibodies (Cell Signaling Technology, Beverly, MA, USA).

ECL results were scanned and the protein bands were quantified using Image J analysis software (National Institutes of Health, USA). Histograms were generated by normalizing the amount of each protein to the total ERK level detected in the same extracted sample. Each experiment was repeated three times. The data presented here were from one representative experiment.

### 2.5. siRNA and Plasmids

T24 cells were transfected using siRNA transfection regent (Qiagen, Hilden, Germany) with 10 nM* ERβ* or* GPPR30* siRNA (Qiagen, Hilden, Germany) according to the manufacturer's instructions; negative siRNA (Qiagen, Hilden, Germany) was used as a negative control. The target sequence of the used* ERβ* siRNA was 5′-CAGCGATTACGCATCGGGATA-3′, and the sequence of used* GPR30* siRNA was 5′-CGGCCACGTCATGTCTCTAAA-3′. After culturing in phenol-red-free RPMI 1640 medium containing 10% dextran-coated charcoal-treated FBS for 24 h, E2 was added to 6-well plates for the qPCR and western blot experiments or to 96-well plates for the MTT assays.

Mammalian expression vectors encoding* ERβ* or* GPPR30 *were constructed by inserting PCR-amplified fragments into pcDNA3 (Invitrogen). Lipofectamine 2000 reagent was used for transfections according to the standard protocols (Invitrogen).

### 2.6. Data Analysis and Statistical Methods

Results from three independent experiments were analyzed using standard error of the mean (SEM). The comparison among groups was analyzed by one-way ANOVA. Values of *P* < 0.05 were considered statistically significant and values of *P* < 0.01 were considered highly significant. All of the statistical analysis was performed using SPSS for Windows Release 13.0 (SPSS, Chicago, IL, USA).

## 3. Results 

### 3.1. T24 Cell Proliferation Was Promoted by E2

**Figure 1:**
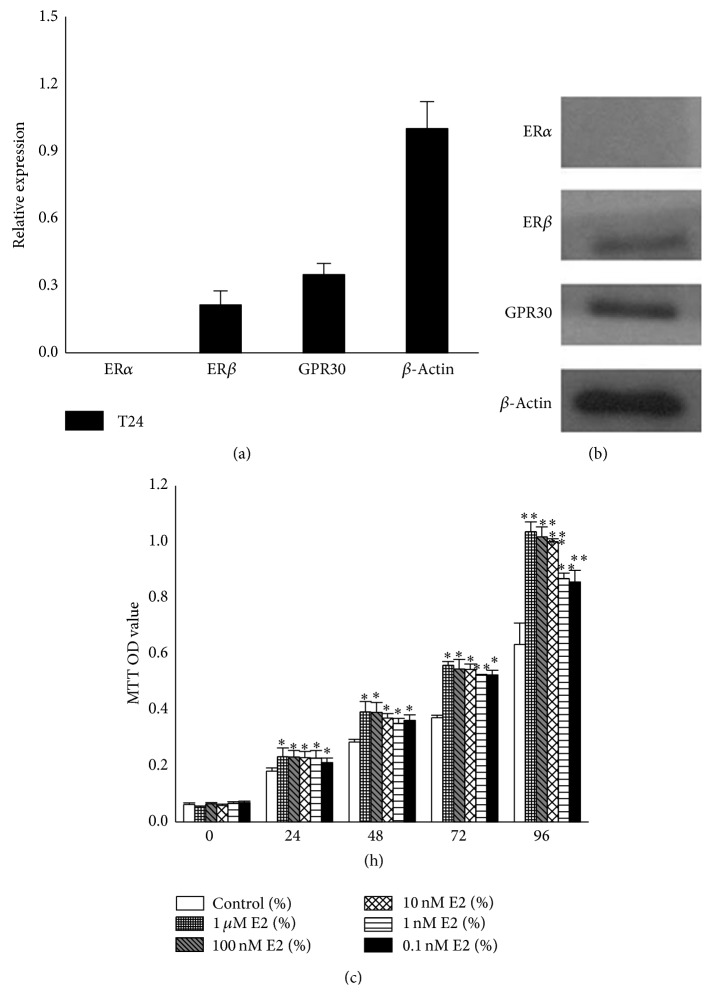
Proliferation of T24 cell was promoted by E2. (a) qPCR analysis of expression of estrogen receptor in T24 cells.* ERα* mRNA was rarely expressed in the cells, and the relative expression levels of* ERβ* and* GPR30* were 0.21 and 0.35, respectively (normalization to *β-actin*). (b) Expression of estrogen receptors in human T24 bladder cancer cells. Twenty micrograms of whole protein extracts was used for western blot analysis. ER*α* was not detected. (c) Cell proliferation promoted by E2. T24 cells were seeded in 96-well plates at a density of approximately 2 × 10^3^ for each well and incubated with E2; then the OD values were examined after 0, 24, 48, 72, and 96 h by MTT assays. 0.1% DMSO was used as the negative control. The values represent the mean ± SD of the data from three independent experiments. ^∗^ *P* < 0.05; ^∗∗^ *P* < 0.01.

To investigate the biological function of E2 in T24 cells, we first explored the expression of estrogen receptors using qPCR and western blotting, which showed that T24 cells expressed* ERβ* and* GPR30* but not* ERα* (Figures 1(a) and 1(b)). To better understand the exact effect of E2 on T24 cells, we incubated the cells with increasing concentrations of E2: 0.1 nM, 1 nM, 10 nM, 100 nM, and 1 *μ*M. Cell proliferation was examined after 0 h, 24 h, 48 h, 72 h, and 96 h using MTT assays, in which the absorbance of formazan indirectly reflected cell activity and cell numbers (Figure 1(c)). These data demonstrated that E2 stimulated T24 cell proliferation in a dose- and time-dependent manner. We selected 10 nM E2 for subsequent experiments due to its higher efficiency and lower toxicity.

### 3.2. GPR30 May Mediate the Inhibitory Effect Induced by E2 in T24 Cells

**Figure 2:**
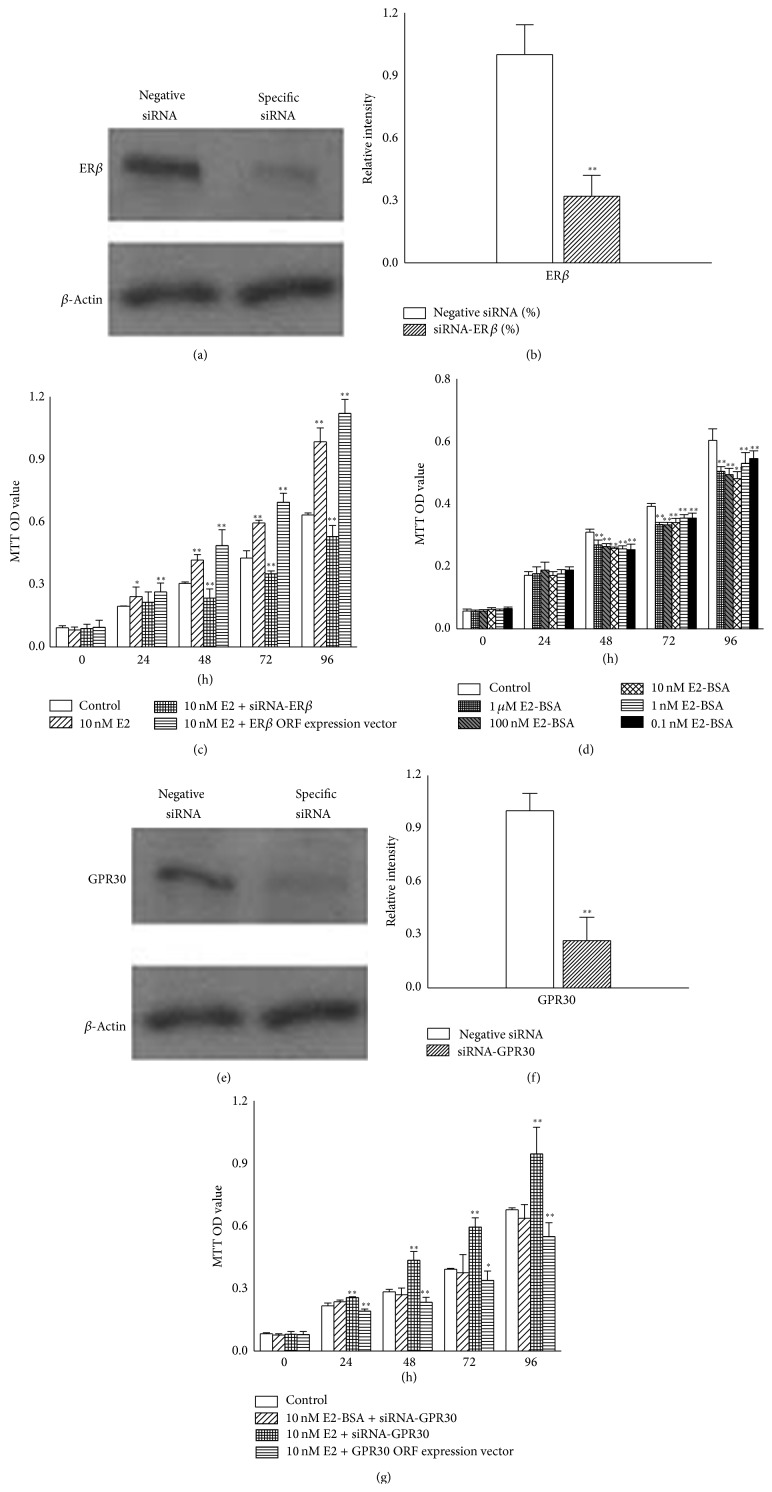
GPR30 mediated an inhibitory effect in T24 cells. (a) T24 cells were transfected with 10 nM specific siRNA against* ERβ*, and then protein expression levels were measured by western blot. (b)* ERβ* mRNA expression levels by qPCR analysis. The level of* ERβ* in control cells was defined as 1.0. (c) E2 inhibited T24 cell proliferation in the absence of ER*β*. Cells were transfected with siRNA against* ERβ* or* ERβ* ORF expression vector and then treated with 10 nM E2 for 0–96 h. MTT assays were performed to measure the cell activity. (d) Cell proliferation was inhibited by E2-BSA. T24 cells were treated as shown in Figure 1(c), and MTT assays were used to monitor the effect of E2-BSA. (e) T24 cells were transfected with siRNA against* GPR30*. The cells were treated as shown in (a), and protein levels were measured by western blot analysis. (f) Expression levels of* GPR30* mRNA by qPCR analysis. The level of* GPR30* was defined as 1.0. (g)* GPR30* mediated inhibition of T24 cell proliferation. The cells were transfected with 10 nM siRNA against* GPR30 *or* GPR30* ORF expression vector and then treated with 10 nM E2 or 10 nM E2-BSA for 0–96 h. MTT assays were used to detect cell activity. The data presented here is one typical experiment from three independent experiments. ^∗^ *P* < 0.05; ^∗∗^ *P* < 0.01.

T24 cells express ER*β* and GPR30, but it was not known exactly which receptor mediated the cell proliferation stimulated by E2. Cells were transfected with siRNA against* ERβ* (Figures 2(a) and 2(b)) or* ERβ* ORF expression vector, and the effect of 10 nM E2 on proliferation was investigated using MTT assays (Figure 2(c)). Surprisingly, cell proliferation was inhibited at 48 h (*P* < 0.01) when cells were transfected with* ERβ* siRNA. After incubation for 96 h, the inhibition rate was increased to 16.58% (*P* < 0.01). However, upregulated cell proliferation was observed from 24 h (*P* < 0.05) in the cells that were only treated with 10 nM E2, and this effect was time-dependent (Figure 2(c)). In contrast, cell proliferation was further promoted by E2 in cells overexpressing* ERβ* (Figure 2(c)).

It has previously been suggested that GPR30 mediates an inhibitory effect in T24 cells [19]. 17*β*-estradiol-17-hemisuccinate-BSA (E2-BSA, Sigma-Aldrich, St. Louis, MO, USA) is too large to pass through the cell plasma membrane. Thus, it could be considered that E2-BSA binds to GPR30, which is localized on the plasma membrane. To validate the biological effect of E2-BSA mediated by GPR30 in T24 cells, the cells were treated with 0.1 nM, 1 nM, 10 nM, 100 nM, and 1 *μ*M E2-BSA for 0–96 h, and MTT assays were performed to measure the cell numbers (Figure 2(d)). The inhibition rate of T24 cells was about 18.06% at 48 h (*P* < 0.01), and the inhibition rate reached 20.38% at 96 h when the cells were treated with 10 nM E2-BSA (*P* < 0.01). Next, we silenced or overexpressed* GPR30* in T24 cells (Figures 2(d) and 2(f)), followed by 10 nM E2-BSA-treatment or 10 nM E2-treatment for 0–96 h (Figure 2(g)). No significant difference was observed between cells treated with 10 nM E2-BSA and control cells, perhaps because E2-BSA can not bind to a receptor. Cell proliferation was promoted when the* GPR30-*silenced cells were treated with 10 nM E2 (*P* < 0.01). In contrast, cell proliferation was further inhibited by E2 in cells overexpressing* GPR30*. Thus, we concluded that the E2 could inhibit cell proliferation in the presence of GPR30 and promoted cell proliferation in other circumstances. This finding may indicate that GPR30 mediated an inhibitory effect on T24 cell proliferation.

### 3.3. Either ER*β* Or GPR30 Mediated Phosphorylation of ERK Induced by E2 through the EGFR-MAPK Pathway

**Figure 3:**
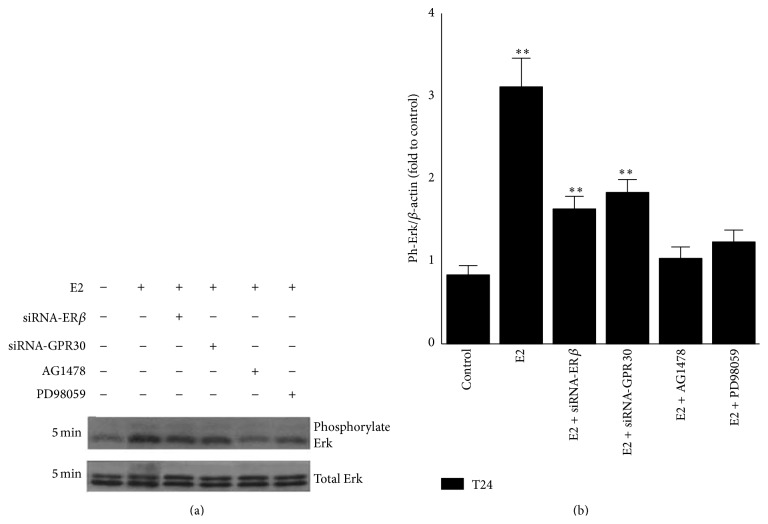
E2 induced activation of ERK through ER*β*/GPR30-regulated EGFR-MAPK pathway in T24 cells. (a) E2 rapidly induced activation of ERK in T24 cells. Cells were transfected with specific siRNA against* ERβ* or* GPR30*, or pretreated with 100 nM AG1478 or 20 *μ*M PD98059 for 30 min. Then 10 nM E2 was added and phosphorylated and total ERK levels were measured by western blot analysis. (b) Histogram of phosphorylation of ERK. The values were normalized to total ERK for each sample. The control was defined as 1.0. Blots are representative of three independent experiments with similar results. ^∗∗^ *P* < 0.01.

Estrogens can generate a rapid nongenomic effect via second messengers, such as G protein, and then activate various downstream kinases such as ERK in cancer cells [21]. Our study showed that phosphorylation of ERK in T24 cells could be rapidly induced after treatment with E2 for 5 min (Figure 3). To evaluate which estrogen receptor (ER*β* or GPR30) was involved in this response, T24 cells were transfected with specific siRNAs against* ERβ* or* GPR30* and incubated for 24 h. Then the phosphorylation of ERK was monitored after treatment with 10 nM E2 for 5 min. Although total levels of ERK were not changed by E2 in both the presence or absence of related siRNAs (*P* < 0.01), the extent of phosphorylated ERK was reduced when* ERβ* or* GPR30* was silenced (Figure 3). This suggests that ERK phosphorylation was mediated by either ER*β* or GPR30 and that there may be a cross talk between the two receptors. When the cells were pretreated with the EGFR (epidermal growth factor receptor) antagonist AG1478 (100 nM) or the MAPK antagonist PD98059 (20 *μ*M) for half an hour, this effect induced by E2 was also blocked (Figure 3). These results indicated that EGFR and MAPK were required for phosphorylation of ERK. We proposed that either ER*β* or GPR30 could mediate the phosphorylation of ERK induced by E2 and hypothesized that activation of ERK in this context was mediated by the EGFR-MAPK pathway via cross talk between ER*β* and GPR30.

### 3.4. E2 Altered the Expression Levels of Relative mRNAs in T24 Cells

**Figure 4:**
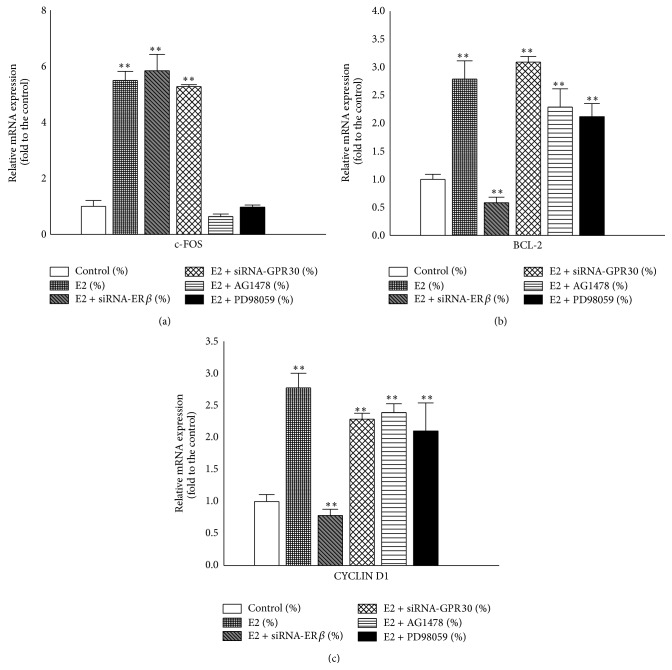
Relative expression levels of genes in T24 cells after treatment with E2. ((a)–(c)) Cells were pretreated as described in Figure 3(a) and then incubated with 10 nM E2 for 48 h. Total RNA was extracted for qPCR.* c-FOS*,* BCL-2*, and* CYCLIN D1* mRNA expression levels were evaluated and normalized to *β-actin* level. The values represent the mean ± SD of the data from three independent experiments. ^∗∗^ *P* < 0.01.

As described above, E2 transduced signals through rapid activation of ERK (Figure 3). According to our hypothesis, this response could involve the activation of both ER*β* and GPR30.* c-FOS* is one of the target genes in the estrogen response [22, 23] and participates in the regulation of cell cycle [24]. BCL-2 is closely associated with apoptosis [25, 26] and CYCLIN D1 is an essential cell cycle regulatory molecule. Therefore, we evaluated the mRNA expression levels of these targets using qPCR after normalization against *β-actin* levels (Figure 4). After treatment with 10 nM E2 for 48 h, the expression levels of* c-FOS*,* BCL-2*, and* CYCLIN D1 *mRNA were 5.5-, 2.8-, and 2.7-fold higher than that of the control, respectively (Figure 4). However,* BCL-2 *and* CYCLIN D1* expression levels were inhibited when the cells were transfected with* ERβ* siRNA (Figures 4(b) and 4(c)), with inhibition rates of 42% and 22%, respectively. In contrast, E2 increases the expression levels of these genes in* GPR30*-silenced T24 cells. These results indicated that ER*β* mediated cell proliferation and GPR30 mediated cell growth inhibition. Furthermore,* BCL-2 *and* CYCLIN D1 *gene expression levels were increased in the presence of EGFR antagonist and MAPK antagonist, suggesting that the cell proliferation promoted by E2 may be independent of the EGFR-MAPK pathway.

### 3.5. E2 Promoted T24 Cell Proliferation Independent of the EGFR-MAPK Pathway

**Figure 5:**
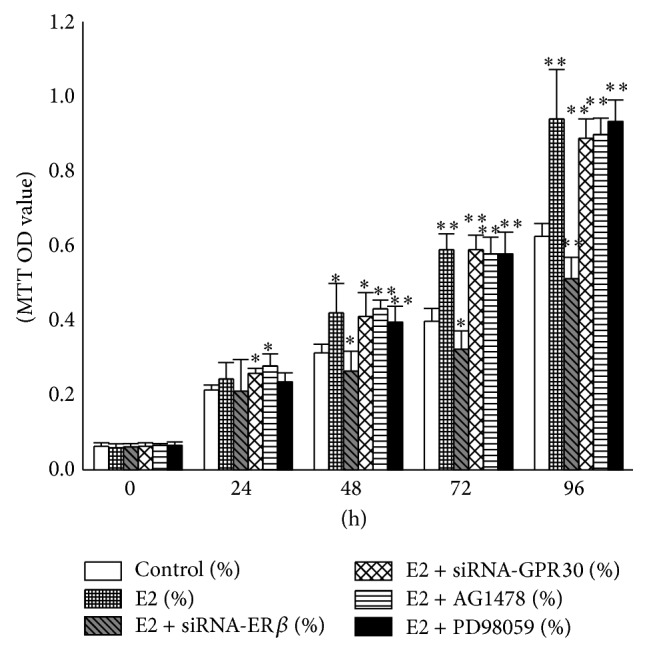
E2 promoted T24 cell proliferation independent of the EGFR-MAPK pathway. T24 cells were pretreated as described in Figure 3. Cells were seeded in 96-well plates at a density of 2 × 10^3^ cells/well and incubated with 10 nM E2. OD values were measured after 0, 24, 48, 72, and 96 h. The values presented here are representative of three independent experiments. ^∗^ *P* < 0.05; ^∗∗^ *P* < 0.01.

To further confirm the molecular mechanisms induced by E2 in T24 cells, we performed additional MTT assays (Figure 5). The data showed that 10 nM E2 stimulated proliferation of T24 cell but inhibited the proliferation of* ERβ*-silenced T24 cells. Furthermore, the proliferation of T24 cells was not affected by 100 nM AG1478 or 20 *μ*M PD98059 in the presence of E2. This experiment provided evidence that there may be cross talk between ER*β* and GPR30, the expression levels of which may determine the cellular responses to E2. ER*β* may play key roles in general response of T24 cells to E2 when the function of GPR30 was weakened or even lost. Finally, the cell proliferation stimulated by E2 was probably independent of the EGFR-MAPK pathway.

## 4. Discussion 

Estrogens, particularly 17*β*-estradiol (E2), are widely acknowledged to be potent regulators of cell proliferation in tissues. Estrogens mediate their effects in target tissues through ERs, and ERs were found to be expressed in most cancer cells. Some studies demonstrated that ER*α* is required for carcinogenesis of the mammary gland [27, 28] and prostate [29]. Reports also suggested that ER*α* contributes to the stimulation of cell proliferation. For instance, ER*α* mediates the induction of breast cancer cell proliferation [30] and the promotion of cell proliferation of ovarian cancer [31] and bladder cancer [5]. However, ER*β* has been observed to exert an inhibitory effect on cell proliferation [30, 32, 33]. In our study, we aimed to investigate the effects mediated by ERs in response to estrogens in bladder cancer. Previous reports published contradictory results regarding the expression levels of ER*α* in bladder cancer cells. Teng et al. reported that the expression of ER*α* in human bladder tumor cells was significantly higher than that in bladder urothelial cells [4]. However, Shen et al. and Tuygun et al. only found weak expression levels of ER*α* in the tumor samples of hundreds of patients [5, 10, 18]. It has also been reported that bladder urothelial cells [4] and tumor cells [4, 5] express equally high levels of ER*β*, suggesting that ER*β* plays more crucial roles in urothelial and bladder cancer cells. Our results are not in agreement with studies, in which the results showed that ER*α* is expressed at high levels in T24 cells and that E2 induced cell proliferation in the absence of* ERβ* [4]. Here, we found that T24 cells expressed ER*β* but rarely ER*α*, and proliferation was stimulated by E2. It was interesting to note that these results were not consistent with the view that ER*β* has an inhibitory effect on cancer cells. Therefore, there may be other receptors involved in this function.

GPR30 is a novel membrane ER [13] and potentially mediates rapid E2-dependent cancer cell proliferation [15, 16, 34, 35]. Our findings suggest that GPR30 may be involved in promoting T24 cell proliferation induced by E2. The proliferation of cells transfected with siRNA against* ERβ* was inhibited in the presence of E2. We considered that nuclear ER*β* may play a key role in the cell proliferation stimulated by E2, and we hypothesized that GPR30 mediated an inhibitory effect in T24 cells. Cell proliferation was stimulated by E2 when* GPR30* was silenced, providing evidence that nuclear ER*β* and GPR30 had opposing effects on cell proliferation in T24 cells: nuclear ER*β* mediated promotion of T24 cell proliferation and GPR30 mediated cell growth inhibition. We considered that the action of ER*β* in response to estrogens should not be generally extrapolated to all tissues.

Here, we found that nuclear ER*β* binding protein E2 stimulated T24 cell proliferation in parallel with immediate phosphorylation of ERK. Either GPR30 or ER*β* can mediate the rapid activation of ERK [36–38]. We examined the activation of ERK induced by E2 after the cells were transfected with siRNA against* GPR30* and found that the extent of ERK phosphorylation was reduced compared to that of the control cells. Furthermore, MTT assays indicated that the effects induced by E2 were reversed when* ERβ* and* GPR30* were silenced. Thus, nuclear ER*β* may play a key role in the response to E2 and was not activated by GPR30 in T24 cells. In some cases, for instance, when* ERβ* was silenced, GPR30 could exert its function to mediate inhibition of cell proliferation.

*c-FOS* gene is a protooncogene upregulated by numerous stimuli that enhance its expression and interaction with c-JUN to form heterodimers to regulate cell proliferation and differentiation [12]. In our study, 10 nM E2 increased* c-FOS* gene expression through the EGFR-MAPK pathway when either of the two receptors was knocked down. Hence, we considered that both receptors could mediate* c-FOS* gene expression. BCL-2 protein is known to regulate apoptosis [25, 26] and normally results in the promotion of tumor cell survival by blocking programmed cell death. Here, E2-induced T24 cell proliferation was associated with an increase in* BCL-2* expression. CYCLIN D1 reflects the G1 to S phase transition in cell cycle, and it plays a specific role in mitosis [39]. We found the expression level of CYCLIN D1 mRNA was more than two-fold higher than that of the control. However, the results were inconsistent with those observed by Teng et al. When* GPR30* was silenced [4], Teng et al. found that E2 increased CYCLIN D1 mRNA levels in T24 cells [19] but did not increase its expression level in the absence of* GPR30* [19]. These two views are incompatible because ER*β* can also mediate the expression of this gene. In our study, this gene was expressed at significantly higher levels in the absence of either ER*β* or GPR30. We supposed that E2 increased the expression of the* c-FOS* gene, and the resulting c-FOS/c-JUN heterodimers increased the expression of the* BCL-2* gene to protect the cells from apoptosis. The c-FOS/c-JUN heterodimers could also increase the relative gene expression, such as CYCLIN D1, which resulted in promoting cell proliferation. Barkhem et al. hypothesized that the long-term effects of estrogens may be mediated by both ER*α* and ER*β* through alterations of gene expression and protein synthesis [40]. In our study, we presumed that the cross talk between nuclear ER*β* and GPR30 mediated E2-promoted T24 cell proliferation. Nuclear ER*β* mainly performed the genomic action, and GPR30 assisted it to execute this response.

Our data indicated that the cell proliferation promoted by E2 was independent of the EGFR-MAPK pathway, because the inhibition of EGFR or MAPK by specific inhibitors could not abolish E2-stimulation of T24 cell proliferation. Silencing of* ERβ* or* GPR30* did not inhibit ERK activation. GPR30 could transactivate EGFR in response to E2 and then induced ERK phosphorylation [30]. And, according to previous reports, ER*β* could also lead to rapid activation of ERK [36, 37]. The activation of ERK was probably not correlated with the cell proliferation in the presence of both nuclear ER*β* and GPR30, and the antagonists of the EGFR-MAPK pathway blocked ERK activation but did not inhibit the cell proliferation stimulated by E2. We presumed that this cell proliferation was possibly mediated by nuclear ER*β* through other pathways, which will be the focus of our future work. GPR30 probably did not exert the key roles in the cells unless its expression level or the ratio of the two receptors reached a crucial level.

## 5. Conclusions 

Our data provide evidence that E2 could stimulate the proliferation of T24 cells. ER*β* and GPR30 receptors can affect EGFR-MAPK/ERK activation, but this stimulation is independent of cell proliferation. ER*β* promoted cell proliferation, while GPR30 inhibited cell proliferation. Since the function of GPR30 is weakened or lost, ER*β* may play the main roles in response to E2 in T24 cells. This study suggests new insights in the understanding of bladder cancer and indicates that ER*β* and GPR30 might be potential new targets for bladder cancer therapy.

## References

[1] Jemal A., Bray F., Center M. M., Ferlay J., Ward E., Forman D. (2011). Global cancer statistics. *Cancer Journal for Clinicians*.

[2] Miyamoto H., Yang Z., Chen Y.-T. (2007). Promotion of bladder cancer development and progression by androgen receptor signals. *Journal of the National Cancer Institute*.

[3] Wu J.-T., Han B.-M., Yu S.-Q., Wang H.-P., Xia S.-J. (2010). Androgen receptor is a potential therapeutic target for bladder cancer. *Urology*.

[4] Teng J., Wang Z.-Y., Jarrard D. F., Bjorling D. E. (2008). Roles of estrogen receptor *α* and *β* in modulating urothelial cell proliferation. *Endocrine-Related Cancer*.

[5] Shen S. S., Smith C. L., Hsieh J.-T. (2006). Expression of estrogen receptors-*α* and -*β* in bladder cancer cell lines and human bladder tumor tissue. *Cancer*.

[6] Wolpert B. J., Amr S., Ezzat S. (2010). Estrogen exposure and bladder cancer risk in Egyptian women. *Maturitas*.

[7] Davis-Dao C. A., Henderson K. D., Sullivan-Halley J. (2011). Lower risk in parous women suggests that hormonal factors are important in bladder cancer etiology. *Cancer Epidemiology Biomarkers and Prevention*.

[8] Tsai M.-J., O'Malley B. W. (1994). Molecular mechanisms of action of steroid/thyroid receptor superfamily members. *Annual Review of Biochemistry*.

[9] Govind A. P., Thampan R. V. (2003). Membrane associated estrogen receptors and related proteins: localization at the plasma membrane and the endoplasmic reticulum. *Molecular and Cellular Biochemistry*.

[10] Bolenz C., Lotan Y., Ashfaq R., Shariat S. F. (2009). Estrogen and progesterone hormonal receptor expression in urothelial carcinoma of the bladder. *European Urology*.

[11] Thomas P., Dong J. (2006). Binding and activation of the seven-transmembrane estrogen receptor GPR30 by environmental estrogens: a potential novel mechanism of endocrine disruption. *The Journal of Steroid Biochemistry and Molecular Biology*.

[12] Filardo E. J., Quinn J. A., Bland K. I., Frackelton A. R. (2000). Estrogen-induced activation of Erk-1 and Erk-2 requires the G protein-coupled receptor homolog, GPR30, and occurs via trans-activation of the epidermal growth factor receptor through release of HB-EGF. *Molecular Endocrinology*.

[13] Revankar C. M., Cimino D. F., Sklar L. A., Arterburn J. B., Prossnitz E. R. (2005). A transmembrane intracellular estrogen receptor mediates rapid cell signaling. *Science*.

[14] Dong S., Terasaka S., Kiyama R. (2011). Bisphenol A induces a rapid activation of Erk1/2 through GPR30 in human breast cancer cells. *Environmental Pollution*.

[15] Albanito L., Madeo A., Lappano R. (2007). G protein-coupled receptor 30 (GPR30) mediates gene expression changes and growth response to 17*β*-estradiol and selective GPR30 ligand G-1 in ovarian cancer cells. *Cancer Research*.

[16] Chan Q. K. Y., Lam H.-M., Ng C.-F. (2010). Activation of GPR30 inhibits the growth of prostate cancer cells through sustained activation of Erk1/2, c-jun/c-fos-dependent upregulation of p21, and induction of G2 cell-cycle arrest. *Cell Death and Differentiation*.

[17] Chevalier N., Bouskine A., Fenichel P. (2011). Role of GPER/GPR30 in tumoral testicular germ cells proliferation. *Cancer Biology and Therapy*.

[18] Tuygun C., Kankaya D., Imamoglu A. (2011). Sex-specific hormone receptors in urothelial carcinomas of the human urinary bladder: a comparative analysis of clinicopathological features and survival outcomes according to receptor expression. *Urologic Oncology: Seminars and Original Investigations*.

[19] Teng J., Wang Z. Y., Prossnitz E. R., Bjorling D. E. (2008). The G protein-coupled receptor GPR30 inhibits human urothelial cell proliferation. *Endocrinology*.

[20] Castelao J. E., Yuan J.-M., Skipper P. L. (2001). Gender- and smoking- related bladder cancer risk. *Journal of the National Cancer Institute*.

[21] Bulayeva N. N., Watson C. S. (2001). Xenoestrogen-induced ERK-1 and ERK-2 activation via multiple membrane-initiated signaling pathways. *Environmental Health Perspectives*.

[22] Maggiolini M., Vivacqua A., Fasanella G. (2004). The G protein-coupled receptor GPR30 Mediates c-fos up-regulation by 17*β*-estradiol and phytoestrogens in breast cancer cells. *Journal of Biological Chemistry*.

[23] Singleton D. W., Feng Y., Burd C. J., Khan S. A. (2003). Nongenomic activity and subsequent c-fos induction by estrogen receptor ligands are not sufficient to promote deoxyribonucleic acid synthesis in human endometrial adenocarcinoma cells. *Endocrinology*.

[24] Shaulian E., Karin M. (2002). AP-1 as a regulator of cell life and death. *Nature Cell Biology*.

[25] Hockenbery D. M. (1995). Bcl-2, a novel regulator of cell death. *BioEssays*.

[26] Kroemer G. (1997). The proto-oncogene Bcl-2 and its role in regulating apoptosis. *Nature Medicine*.

[27] Miermont A. M., Parrish A. R., Furth P. A. (2010). Role of ER*α* in the differential response of Stat5a loss in susceptibility to mammary preneoplasia and DMBA-induced carcinogenesis. *Carcinogenesis*.

[28] Yoshidome K., Shibata M. A., Couldrey C., Korach K. S., Green J. E. (2000). Estrogen promotes mammary tumor development in C3(1)/SV40 large T-antigen transgenic mice: paradoxical loss of estrogen receptoralpha expression during tumor progression. *Cancer Research*.

[29] Ellem S. J., Risbridger G. P. (2007). Treating prostate cancer: a rationale for targeting local oestrogens. *Nature Reviews Cancer*.

[30] Helguero L. A., Faulds M. H., Gustafsson J.-Å., Haldosén L.-A. (2005). Estrogen receptors alfa (ER*α*) and beta (ER*β*) differentially regulate proliferation and apoptosis of the normal murine mammary epithelial cell line HC11. *Oncogene*.

[31] Yang J., Wang Y., Gao Y., Shao J., Zhang X. J., Yao Z. (2009). Reciprocal regulation of 17*β*-estradiol, interleukin-6 and interleukin-8 during growth and progression of epithelial ovarian cancer. *Cytokine*.

[32] Schleipen B., Hertrampf T., Fritzemeier K. H. (2011). ER*β*-specific agonists and genistein inhibit proliferation and induce apoptosis in the large and small intestine. *Carcinogenesis*.

[33] Pinton G., Thomas W., Bellini P. (2010). Estrogen receptor *β* exerts tumor repressive functions in human malignant pleural mesothelioma via EGFR inactivation and affects response to Gefitinib. *PLoS ONE*.

[34] Vivacqua A., Bonofiglio D., Albanito L. (2006). 17*β*-Estradiol, genistein, and 4-hydroxytamoxifen induce the proliferation of thyroid cancer cells through the G protein-coupled receptor GPR30. *Molecular Pharmacology*.

[35] Smith H. O., Arias-Pulido H., Kuo D. Y. (2009). GPR30 predicts poor survival for ovarian cancer. *Gynecologic Oncology*.

[36] Filardo E. J., Thomas P. (2005). GPR30: a seven-transmembrane-spanning estrogen receptor that triggers EGF release. *Trends in Endocrinology & Metabolism*.

[37] Watson C. S., Alyea R. A., Jeng Y.-J., Kochukov M. Y. (2007). Nongenomic actions of low concentration estrogens and xenoestrogens on multiple tissues. *Molecular and Cellular Endocrinology*.

[38] Kim J.-H., Jeong I.-Y., Lim Y., Lee Y. H., Shin S. Y. (2011). Estrogen receptor *β* stimulates Egr-1 transcription via MEK1/Erk/Elk-1 cascade in C6 glioma cells. *BMB Reports*.

[39] Sherr C. J., Roberts J. M. (2004). Living with or without cyclins and cyclin-dependent kinases. *Genes and Development*.

[40] Barkhem T., Nilsson S., Gustafsson J.-Å. (2004). Molecular mechanisms, physiological consequences and pharmacological implications of estrogen receptor action. *American Journal of PharmacoGenomics*.

